# Unique Challenges of Atopy Treatment in the Correctional Facility System: A Case Study

**DOI:** 10.7759/cureus.5395

**Published:** 2019-08-16

**Authors:** Justin Chin, Koji Ota, Lauren Strazzulla, Catherine Mills, Mary Lee Wong

**Affiliations:** 1 Family Medicine, Touro College of Osteopathic Medicine, New York, USA; 2 Internal Medicine, Mount Sinai Beth Israel Medical Center, New York, USA; 3 Allergy and Immunology, Mount Sinai Beth Israel Medical Center, New York, USA

**Keywords:** conjugal visit, atopy, allergy, correctional facility, anaphylaxis, allergy testing, prison, jail, incarcerated population, incarceration

## Abstract

The United States houses one of the largest populations of incarcerated individuals in the world. By extension, the healthcare needs of incarcerated individuals are molded by the unique environmental and institutional circumstances that are less often a concern for the general nonincarcerated community. Conjugal visits pose a distinctive challenge for administration and physicians alike as this presents an intersection between the isolated, controlled correctional facility system and the broader, outside world. Here we present a case of severe urticaria and anaphylaxis associated with a patient’s conjugal visits as well as the challenges in management and treatment of atopy relative to the correctional facility system.

## Introduction

The United States has one of the highest incarceration rates in the world, with over two million (0.7%-0.8%) of its population in local, state, and federal correctional facilities [1]. From prisons and jails to probation and parole, the effects of incarceration extend well beyond the offender, and can impact the health and well-being of their families, friends, and communities. Incarceration rates have increased over the past 40 years because of progressively stricter state and federal sentencing policies, peaking in the past decade [2]. Subsequently, the healthcare needs to those who interface with correctional facility have also grown, with age, gender, and race creating multifactorial challenges [3]. As more Americans become incarcerated, physicians have begun to witness how the correctional facility system has created unique health issues and complications that extend beyond its physical walls [4].

Few studies have specifically examined the effect of environmental conditions found in correctional facilities on allergic and dermatologic diseases [3, 5-6]. Even less have examined how correctional facilities can affect nonincarcerated people who interact frequently with the system, as in the case of conjugal visits and support staff [6]. Also known as the Family Reunion Program (FRP) in New York (one of four states that still conducts this type of program), conjugal visits allow inmates and their families to interact in a nonprison setting, with the goal of preserving the family unit. In doing so, the program aims to facilitate reintegration into the family and community once released, which reduces the likelihood of recidivism [7]. Here we present a case of severe urticaria and anaphylaxis associated with a patient’s conjugal visits as well as the challenges in management and treatment of atopy relative to the correctional facility system.

## Case presentation

A 37-year-old Caucasian female with a past medical history of asthma, environmental and seasonal allergies, and multiple episodes of anaphylaxis presented to the outpatient allergy and immunology clinic for evaluation of severe, recurrent allergic reactions to food and environmental triggers.

Her symptoms first began in November of 2016 during a conjugal visit at an upstate New York correctional facility. At that time, there was an electrical fire at a nearby trailer, which caused smoke to enter the room and resulted in an anaphylactic reaction that was unresponsive to epinephrine injection. Upon arrival to the ED, she required admission intubation and ICU monitoring. Since that time, she has had numerous ER visits and hospitalizations (2-3 per month) and presented each time with dyspneic symptoms secondary to facial, throat, and tongue swelling. Epinephrine injections (EpiPen), oral diphenhydramine (Benadryl), and inhaled albuterol treatments have provided minimal improvements during each episode; ultimately requiring numerous ICU admissions and intravenous epinephrine.

She has not been able to identify a solitary trigger; however, she has attributed her symptoms to various foods such as salad dressing and conjugal visits. She also anecdotally reported that there are shared barbeque grills outside the trailers where conjugal visits occur and that her symptoms worsen when they are in use. During her conjugal visits, she stated that she would also develop diffuse urticaria on her face and body when using sheets supplied by the facility. These rashes have not occurred at home where she uses hypoallergenic sheets washed in fragrance-free detergent. She has been unable to discover the material or handling of the sheets supplied for her conjugal visits. Attempts to bring her own sheets have been denied due to the need for physician documentation.

**Figure 1:**
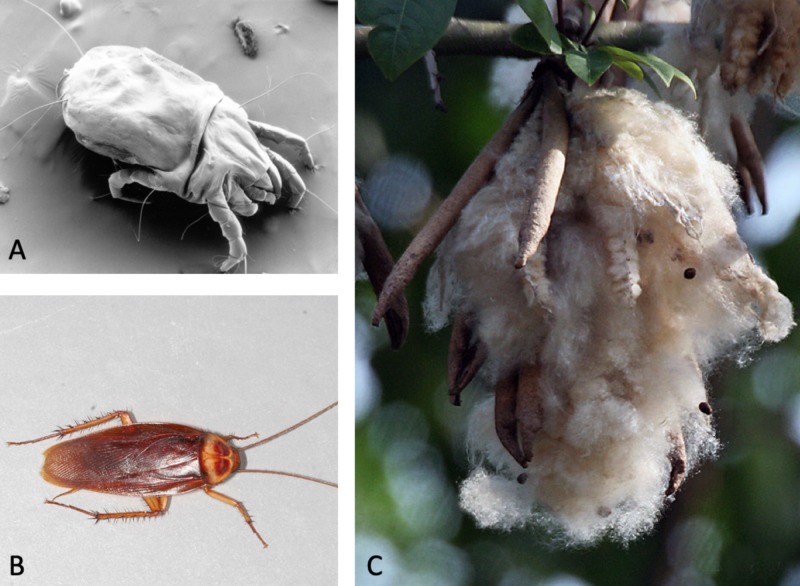
Visual representation of the patient's allergies. A: dust mites; B: cockroach; and C: Kapok fibers (commonly found in mattress/bedding). Original graphic created by Justin Chin.

Environmental allergen skin prick testing during her office visit revealed 2+ reaction to dust mites (including *D. pteronyssinus* and *D. farinae*), kapok fibers and mattress dust, and cockroaches (including *P. americana* and *B. germanica*) (Figure 1). Food allergen skin prick testing showed no skin reactions. Education regarding her results and possible triggers were discussed at length and she was reminded to use an EpiPen if her symptoms became severe. A physician’s note was given with the recommendation that the patient be allowed to bring her own sheets to conjugal visits. The patient was lost to follow-up.

## Discussion

Atopy is the genetic predisposition to produce specific IgE following exposure to allergens and is classically characterized by concomitant or temporally successive presence of atopic dermatitis, allergic rhinitis, and asthma [8]. On a cellular level, atopy occurs via a TH2 response in which CD4+ T helper cells become activated. Once bound to the major histocompatibility complex, the unit produces large quantities of interleukins 4 and 13 [9]. This in turn promotes the allergen-specific IgE production by plasma cells. Subsequently, sensitization and development of true allergies can occur in the atopic march, which describes the natural history or typical progression of allergic diseases [10]. In the general population, studies of atopy and allergies are limited as multiple factors such as the definition of allergy, study populations, methodologies, geographic variation, population age, and difference in food intake can intertwine and affect reporting and quantification [11]. With estimates anywhere between 1% and 10% of the general population, even less is known about atopy and allergies in the incarceration system as it is designated as a vulnerable population by Department of Health and Human Services, which requires greater protections and consent protocols [12].

The environmental conditions that incarcerated individuals are subjected to create unique health challenges for this patient population. In comparing the prevalence of allergic rhinitis in the general population to those living in restricted premises, those who are confined tend to develop allergic rhinitis later in life, have perennial allergic rhinitis instead of seasonal allergic rhinitis, and also have bronchial asthma due to chronic daily exposure to the same allergens [13-14]. Unsurprisingly, certain dermatological complaints and infections, such as atopic dermatitis, tinea pedis, and condyloma acuminatum, are represented at a significantly higher proportions compared with other nonincarcerated populations [14]. These findings have been attributed to various factors such as poor hygiene and ventilation, use of harsh chemical soaps that irritate the skin, and living in high density accommodations for prolonged periods of time. The high rates of inflammatory skin conditions caused by exacerbation of the itch-scratch cycle and resulting in lichenification, suggest that prisoners may be exposed to environmental allergens that are adversely affecting their health and manifesting as allergic and cutaneous symptoms [15]. From a therapeutic standpoint, being in the prison system poses unique challenges in allergy management as common antihistamines and decongestants are regulated due to abuse potential and possible conversion to illicit drugs such as methamphetamines [16].

**Figure 2:**
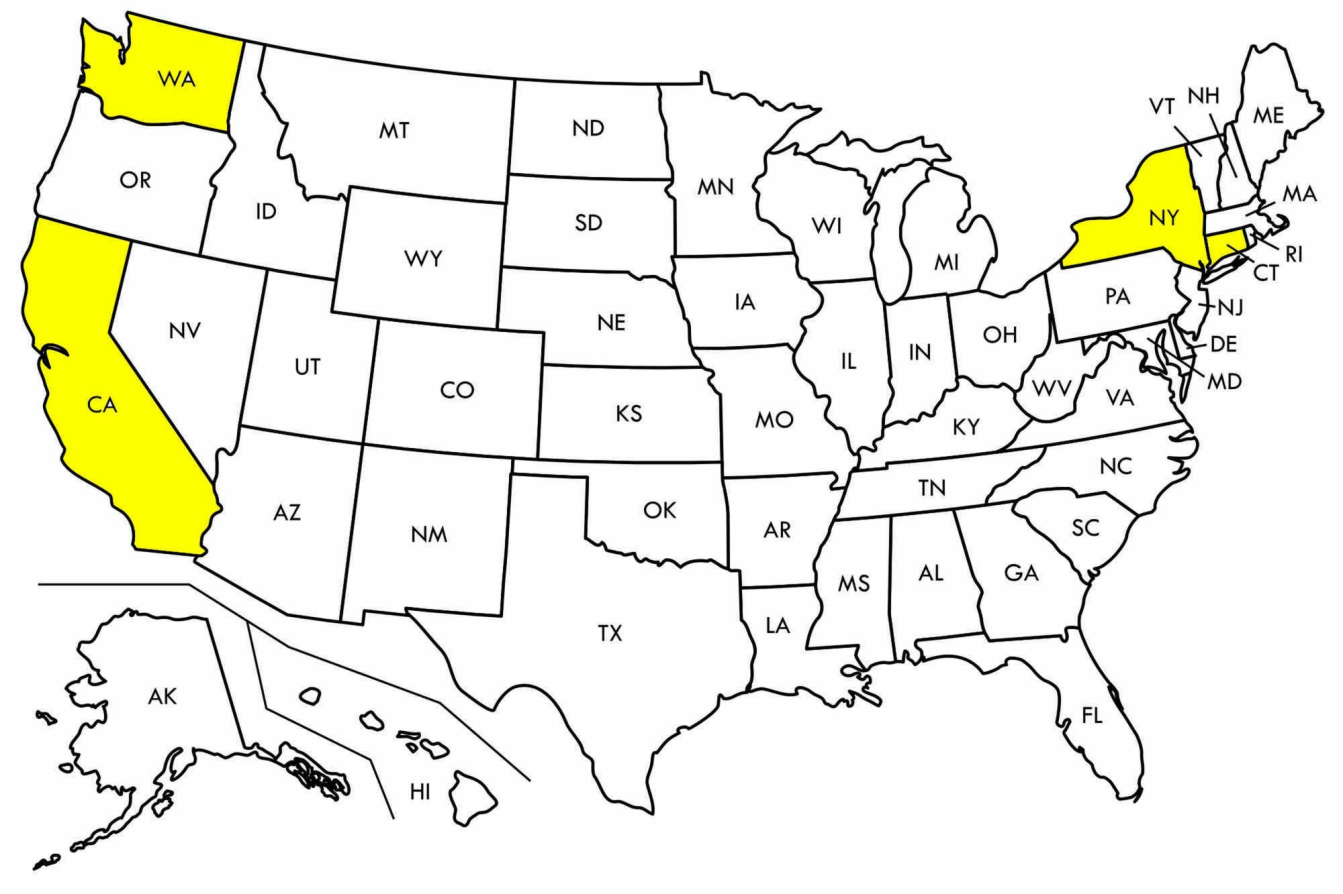
As of 2015, California, Connecticut, New York, and Washington (yellow) are the only four states that allow for conjugal visits. Original graphic created by Justin Chin.

With the limited research on the incarcerated population, conjugal visits are a unique situation in which the insulated nature of the correctional facility system interfaces with the general population. Present in only four states across the United States, numerous federal and state guidelines dictate the specific details of the visits and can vary across facilities (Figure 2) [17]. For the case presented, the patient was in New York, in which conjugal visits are known as the FRP and are regulated by the New York State Department of Corrections and Community Supervision (NYS DOC)[7]. In “text of rule” of the FRP, it has delineated that visitors may only bring a few personal belongings, largely limited to personal hygiene items. The facility is to provide all linens and soaps. FRP’s directive #4500 does include a provision for the compliance of the Americans with Disabilities Act, in allowing for “reasonable accommodations” for disabled inmates and visitors in the program, it is unclear whether this case of urticarial allergies and atopy secondary to unknown linen and detergent usage would qualify as a disability [7]. There are no restrictions in bringing medications such as an EpiPen in the event that the patient develops a severe allergic reaction while at the facility.

As the FRP visits are a privilege, not a right, it seems unlikely that the NYS DOC would approve a visitor bringing his or her own sheets to the prison for a conjugal visit. Additionally, given the rationale behind the abolishment of conjugal visits in many states revolves around safety, it is important to recall that correctional facilities are generally strictly regimented and regulated for the safety of everyone involved [18]. As seen in this case, the easiest solution would be to allow the patient to bring her own sheets as this minimizes the variables associated with triggering an allergic reaction. Without the ability to test the sheets and surroundings for dust and kapok, it is difficult to identify a direct cause and by extension, suggest a definite solution [19]. Due to anticipated costs and the need for administrative approval, other solutions such as placing facilities supplied linens in an additional drying cycle or use of hypoallergenic detergent may not be possible without extensive studies or research [20]. Nevertheless, this case report highlights the need to periodically assess policies in the correctional facility system to ensure that the safety and health of everyone involved. By doing so, a simple change in allowing the patient to bring their own sheets may prevent fatal allergic reactions that could otherwise been easily avoided. 

## Conclusions

Despite increasing rates of incarceration, little is known about how the correctional facility environment can impact the health and healthcare of those that reside and interact with the system. While limited studies exist when reviewing atopic, allergic, and dermatologic health issues endemic to those incarcerated, even less has been done on these problems relative to the general population. Conjugal visits act as a connection between the correctional facility system and the larger nonincarcerated world, thus posing potentially unique healthcare situations. As seen in this case, even though the patient was not directly part of the system, the choices and decisions made by facility administration greatly impacted her health as she participated in conjugal visits. She was faced with severe allergies that were exacerbated when interacting with the correctional facility system, with her multiple ER visits and episodes of anaphylaxis. While simple solutions such as bringing one’s own sheets may be the easiest, it is important to remember that safety and security are paramount for all those involved.
